# Airway smooth muscle inflammation is regulated by microRNA‐145 in COPD

**DOI:** 10.1002/1873-3468.12168

**Published:** 2016-04-19

**Authors:** Lawrence O'Leary, Kenan Sevinç, Ilektra M. Papazoglou, Bernadett Tildy, Karen Detillieux, Andrew J. Halayko, Kian Fan Chung, Mark M. Perry

**Affiliations:** ^1^ Airways DiseaseNational Heart and Lung InstituteImperial CollegeLondonUK; ^2^ Royal Brompton NIHR Biomedical Research UnitLondonUK; ^3^ Departments of Internal Medicine & PhysiologyRespiratory HospitalWinnipegMBCanada; ^4^ Molecular NeurosciencesThe Dubowitz Neuromuscular CentreUCL Institute of Child HealthLondonUK

**Keywords:** COPD, inflammation, microRNA

## Abstract

Chronic obstructive pulmonary disease (COPD) is a common, highly debilitating disease of the airways, primarily caused by smoking. Chronic inflammation and structural remodelling are key pathological features of this disease, in part caused by the aberrant function of airway smooth muscle (ASM) cells under the regulation of transforming growth factor (TGF)‐β. miRNA are short, noncoding gene transcripts involved in the negative regulation of specific target genes, through their interactions with mRNA. Previous studies have proposed that mRNA‐145 (*miR‐145*) may interact with *SMAD3*, an important downstream signalling molecule of the TGF‐β pathway. TGF‐β was used to stimulate primary human ASM cells isolated from healthy nonsmokers, healthy smokers and COPD patients. This resulted in a TGF‐β‐dependent increase in CXCL8 and IL‐6 release, most notably in the cells from COPD patients. TGF‐β stimulation increased *SMAD3* expression, only in cells from COPD patients, with a concurrent increased *miR‐145* expression. Regulation of *miR‐145* was found to be negatively controlled by pathways involving the MAP kinases, MEK‐1/2 and p38 MAPK. Subsequent, overexpression of *miR‐145* (using synthetic mimics) in ASM cells from patients with COPD suppressed IL‐6 and CXCL8 release, to levels comparable to the nonsmoker controls. Therefore, this study suggests that *miR‐145* negatively regulates pro‐inflammatory cytokine release from ASM cells in COPD by targeting *SMAD3*.

## Abbreviations

**ASM**, airway smooth muscle

**COPD**, chronic obstructive pulmonary disease

**CXCL8**, CXC chemokine ligand 8

**HSFBs**, hypertrophic scar fibroblasts

**IL‐6**, interleukin 6

**MAPKs**, mitogen‐activated protein kinases

***miR‐145***, microRNA‐145

**NF‐κB**, nuclear factor‐κB

**SSc**, systemic sclerosis

**TGF**, transforming growth factor

**VSMCs**, vascular smooth muscle cells

**WNT**, wingless/integrase‐1

Chronic obstructive pulmonary disease (COPD) is a chronic inflammatory condition of the lung of high global prevalence 1 and is associated with high morbidity, mortality and socioeconomic cost 2; moreover, its contribution to deaths worldwide is predicted to increase over the course of both the current and next decade 3.

Chronic obstructive pulmonary disease is a heterogeneous condition, primarily affecting the lung, but often with significant systemic features, which can augment the morbidity of the disorder and hamper its management 2. The pulmonary component of the disease refers to a progressive and largely irreversible obstruction of airflow, due to a pathological combination of narrowing of the small airways, parenchymal destruction and structural remodelling 4. The aberrant remodelling occurs in addition, or in response, to the ongoing and worsening inflammation 5. It is associated with architectural alterations to the bronchi 6, small airways and parenchymal tissue and affects the epithelium, its underlying extracellular matrix and the surrounding smooth muscle and these changes have been correlated with disease severity 7. Although the primary cause of COPD in more economically developed countries is cigarette smoking, the majority of heavy smokers do not develop the disease 8 and indicators for determining those that will be affected remain elusive.

An increased ASM mass has been observed in both the large and small airways in COPD 6, which correlates with disease severity 7. Whether increased ASM mass in COPD is due to either hypertrophy or hyperproliferation, or a combination of both has not been definitively determined. ASM cells have also been shown to produce a number of cytokines, chemokines and growth factors in response to a variety of inflammatory stimuli, which may contribute towards the inflammatory process in COPD 9. Among these secreted cytokines are CXC chemokine ligand 8 (CXCL8) and interleukin 6 (IL‐6) both of which have been found to be elevated in the sputum of patients with COPD; even more so during exacerbations 10.

Transforming growth factor (TGF)‐β is a pleiotropic cytokine that stimulates ASM proliferation and pro‐inflammatory cytokine production 11, 12, and elevated TGF‐β secretion by pulmonary epithelial cells from patients with COPD has been observed in comparison to healthy controls 13. TGF‐β signalling is typically through the Smad‐dependent pathway 14 although other signalling pathways can also be involved, including the nuclear factor‐κB (NF‐κB) pathway, pathways involving mitogen‐activated protein kinases (MAPKs) and the Wingless/integrase‐1 (WNT) pathway 15.

In addition to the transcription of mRNA, which forms the first step of the ‘central dogma of molecular biology’, noncoding RNA are transcribed from DNA 16. miRNA bind, in a complementary manner, to the 3′ end of their target mRNA and instigate there suppression/degradation 16. Cell proliferation has been shown to be regulated by microRNA‐221 (*miR‐221*) in ASM cells from patients with asthma 12; in a human epithelial cell line 17; and in murine vascular smooth muscle cells (VSMCs) 18. IL‐6 and CXCL8 secretion has also been shown to be attenuated by miRNA; by *miR‐221* in ASM cells from patients with asthma 12; and by *miR‐146a* and *miR‐146b* in human alveolar epithelial cells 19, 20. Our previous studies have helped to identify *miR‐145* as a potential key regulator of airway smooth muscle function in COPD. Firstly, it is highly expressed in the healthy lung 21 and in healthy ASM cells specifically 22. It has also been shown to be overexpressed in the airways of patients with cystic fibrosis, and to correlate with a decrease in *SMAD3* expression 23. A number of human and animal models have linked *miR‐145* to mechanisms that could also contribute towards the development of COPD 24, 25. Smooth muscle cell proliferation correlated inversely with expression levels of *miR‐145* in murine 26, 27, 28, leporine 29 and human 28 vasculatures. Moreover, exposure to cigarette smoke has been shown to affect expression levels of *miR‐145* in the lungs of rats 30.

We hypothesized that increased IL‐6 and CXCL8 release from the ASM cells of COPD patients is mediated by the TGF‐β–induced expression of *miR‐145*. We examined the effects of TGF‐β upon ASM IL‐6 and CXCL8 release from patients with COPD, and in healthy nonsmokers and healthy smokers. We then examined the regulation of *miR‐145* with specific kinase inhibitors. Finally, we examined the effects of modulating the expression levels of *miR‐145* in these cells on cytokine release and on the phosphorylation of SMAD3. *miR‐145* controls the excessive cytokine release observed in ASM cells from patients with COPD, by reducing SMAD3 phosphorylation.

## Materials and methods

### Primary human ASM cell culture

Primary human ASM cells were previously dissected from the lungs of healthy nonsmokers, healthy smokers and patients with COPD; disease and smoking status were defined according to guidelines produced by the American Thoracic Society 31. Healthy smokers had a smoking history of at least 10 pack years. There were significant differences between FEV_1_ in litres, FEV_1_ percent predicted, and FEV_1_/FVC ratio between smokers and patients with COPD compared with nonsmokers but matched for age and smoking history (Table 1).

**Table 1:** Patient characteristics

	Nonsmokers	Smokers	COPD
*n*	9	9	9
Age (years)	66.4 ± 12.72	59.2 ± 7.6	65.4 ± 6.6
Sex (♂ – ♀)	7 – 2	4 – 5	5 – 5
Pack years smoking	N/A	29.25 ± 3.3	38.32 ± 26.92
FEV_1_ (L)	4.02 ± 0.48	3.12 ± 0.78	1.76 ± 0.45
FEV_1_ (% Predicted)	104.23 ± 7.28	101.5 ± 4.51	77 ± 21.97
FEV_1_/FVC (%)	78.89 ± 5.98	77.57 ± 3.32	38.88 ± 15.75
PC_20_ (mg·mL^−1^)	> 16	> 16	Too severe

FEV_1_, forced expiratory volume in 1 s; FVC, forced vital capacity; PC_20_, provocative concentration of methacholine causing a 20% fall in FEV_1_. Data shown as mean ± SEM.

ASM cells were cultured and plated as previously described 11, 12, 22, 32. ASM cells were plated onto 96‐well plates for the measurement of cytokine release, and six well plates for RNA and protein extraction. Confluent cells were growth‐arrested by FCS deprivation for 24 h in Dulbecco's Modified Eagle's Medium supplemented with sodium pyruvate (1 mm), l‐glutamine (2 mm), nonessential amino acids (1 : 100), penicillin (100 U·mL^−1^)/streptomycin (100 mg·mL^−1^), amphotericin B (1.5 mg·mL^−1^) and BSA (0.1%). Passages 3–4 from nine different donors were used. Cells were stimulated in triplicate ± TGF‐β at the indicated concentrations.

Alternatively, ASM cells were cultured for 1 h in the presence or absence of the indicated concentrations of TPCA‐1 (an IKK‐2 inhibitor), PD098059 (a MEK‐1/2 inhibitor), SP600125 (a JNK‐1/2 inhibitor) and SB 203580 (a p38 MAP kinase inhibitor) and then stimulated with 1 ng·mL^−1^ of TGF‐β for 24 h. All inhibitors were obtained from Calbiochem. The supernatants were removed, and IL‐6 and CXCL8 levels were determined by DuoSet ELISA (R&D Systems, Abingdon, UK).

### miRNA and mRNA Expression

The human (hsa)‐*miR‐145* and *SMAD3* expression levels were measured as previously described 11, 12, 22.

### Transfection with *miR‐145* mimics and controls

ASM cells were transfected as previously described 11, 12. A mimic for *miR‐145* and controls were obtained from Ambion/Applied Biosystems, Ltd. (Paisley, UK). Transfected cells were plated into 96‐well or 6‐well plates, and left to adhere overnight before being serum starved for 6 h before stimulation with 1 ng·mL^−1^ TGF‐β for the indicated times.

### Western blotting

Proteins were measured as previously described 12, 32, 33. Antibodies against human phospho‐S423‐S425‐Smad3 and total Smad3 were purchased from AbCam (Cambridge, UK).

### Data analysis

Data were analysed using graphpad prism, version 5.03 (GraphPad Software, San Diego, CA). Data were not normally distributed (as assessed by the Kolmogorov–Smirnov test), and therefore groups were compared using the Dunn nonparametric test. All data are expressed as means ± SEMs. Significance was defined as a *P* value of less than 0.05.

## Results

### The effect of TGF‐β stimulation on CXCL8 and IL‐6 release and *SMAD3* and *miR‐145* expression by ASM cells after 24 h

**Figure 1:**
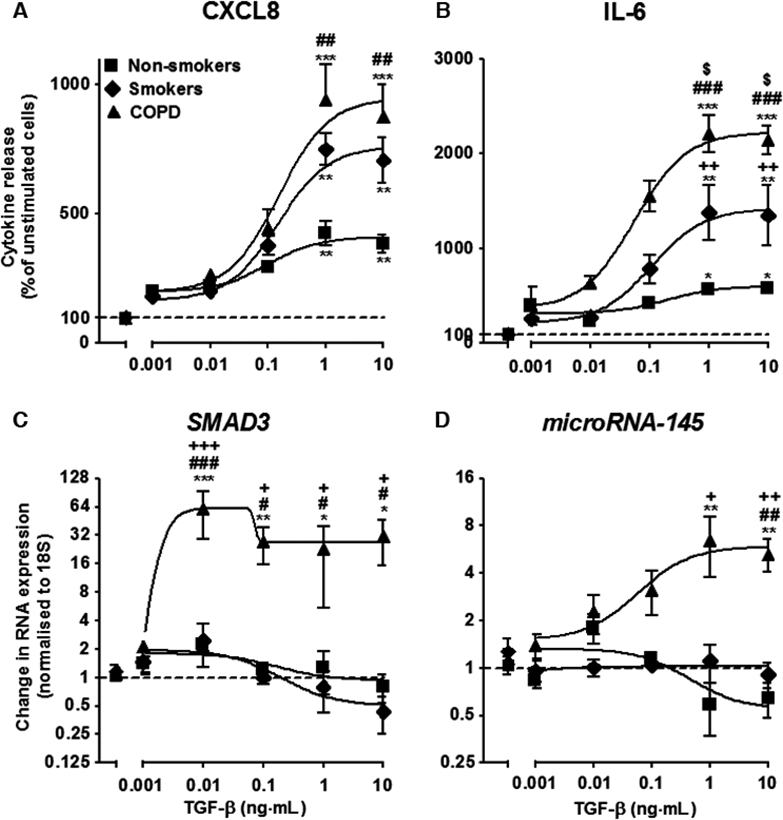
Effect of increasing concentrations of transforming growth factor–β (TGF‐β) on airway smooth muscle (ASM) CXCL8 (A) and IL‐6 release (B), *SMAD3* (C) and *miR‐145* (D) expression from the ASM cells of non‐smokers, smokers and patients with COPD at 24 h. Points represent the means ± SEMs from nine ASM donors in each group. *^/$/#^ *P* < 0.05; **^/$$/##^ *P* < 0.01; ***^/###/$$$^ *P* < 0.001. Asterisks indicate comparison with no TGF‐β control. Hash signs indicate COPD vs. nonsmoker ASM cells. Dollar signs indicate COPD vs. smoker ASM cells. Plus signs indicate smoker vs. nonsmoker ASM cells.

ASM cells were stimulated with 2.5% FCS and TGF‐β at the indicated concentrations (0.001–10 ng·mL^−1^) for 24 h. TGF‐β induced a concentration‐dependent increase in CXCL8 and IL‐6 release from ASM cells which plateaued at 1 ng·mL^−1^ in the nonsmokers (*P* < 0.05), smokers (*P* < 0.01) and COPD (*P* < 0.001) cells (Fig. 1A,B). A significant increase in CXCL8 release was observed in the COPD ASM cells compared to the nonsmokers (*P* < 0.01) when the ASM cells were stimulated with 1 ng·mL^−1^ of TGF‐β (Fig. 1A). Furthermore, there was significant increase in IL‐6 release between the nonsmokers and smokers (*P* < 0.01), nonsmokers and COPDs (*P* < 0.001), and the smoker and COPD ASM cells (*P* < 0.05) (Fig. 1B).

TGF‐β (0.01 ng·mL^−1^) induced an increase in *SMAD3* expression in ASM cells from COPD patients ~ 60‐fold higher than baseline (*P* < 0.001; Fig. 1C). Relatively little change to *SMAD3* expression was seen in the nonsmokers and smokers compared to unstimulated cells. *miR‐145* expression in ASM cells from COPD patients exhibited a concentration dependent increase which plateaued at 1 ng·mL^−1^ (*P* < 0.01) (Fig. 1D). A significant increase in expression was observed in the COPD ASM cells compared to the nonsmokers and smokers (both *P* < 0.01) (Fig. 1D).

### The effects of specific kinase inhibitors on CXCL8 and IL‐6 release by ASM cells stimulated with FCS and TGF‐β after 24 h

In previous studies, we and others have demonstrated that cytokines can induce activation of IKK2/NF‐κB and the MAP kinases, ERK‐1/2, JNK‐1/2 and p38 MAP kinase in ASM cells and that these are inhibited in the presence of the selective pharmacological inhibitors of these 34, 35, 36, 37, 38, 39, 40, 41, 42, 43. We therefore used the biological active concentrations of these inhibitors to examine the role of the NF‐κB and MAP kinases pathways during *miR‐145* expression.

**Figure 2:**
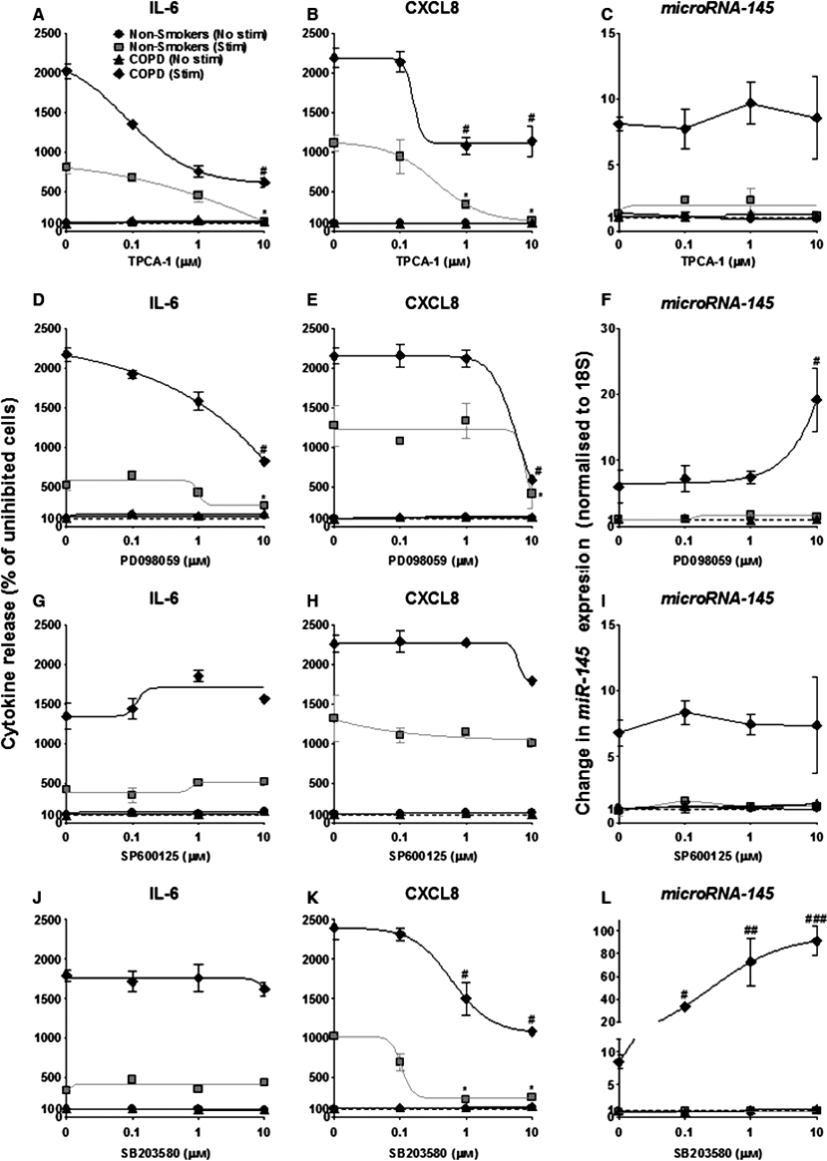
Effect of inhibitors of IKK2 and MAP kinases upon TGF‐β‐induced IL‐6 and CXCL8 release, and *miR‐145* expression from the ASM cells of nonsmokers and patients with COPD at 24 h. ASM cells were pretreated for 60 min with the indicated concentrations of the inhibitors of IKK‐2 (TPCA‐1), MEK‐1/2 (PD098059), JNK‐1/2 (SP600125) and p38 MAP kinase (SB203580). Following exposure to vehicle control or TGF‐β (1 ng·mL^−1^) for 24 h, the release of IL‐6 and CXCL8 was determined by ELISA. *miR‐145* expression was measured by RT‐PCR. Points represent the means ± SEM of nine ASM donors in each group. *^/#^ *P* < 0.05; ^##^ *P* < 0.01; ^###^ *P* < 0.001. Asterisks indicate stimulated nonsmoker comparison with no TGF‐β control. Hash signs indicate stimulated COPD comparison with no TGF‐β control.

Following 1 h pre‐treatment with inhibitors, ASM cells were stimulated with TGF‐β (1 ng·mL^−1^) and the generation of IL‐6 (Fig. 2A,D,G,J), CXCL8 (Fig. 2B,E,H,K) and *miR‐145* (Fig. 2C,F,I,L) were determined at 24 h. Exposure to TPCA‐1 completely inhibited production of IL‐6 and CXCL8 in the non‐smokers at 10 μm, and a significant reduction was observed in the COPD ASM cells (both *P* < 0.05) (Fig. 2A,B). No effect was observed upon *miR‐145* expression (Fig. 2C). The MEK‐1/2 inhibitor (10 μm) also attenuated IL‐6 and CXCL8 production (both *P* < 0.05) (Fig. 2D,E). Interestingly, a significant increase in *miR‐145* expression was observed in the COPD ASM cells (*P* < 0.05) (Fig. 2F). Inhibition of the JNK‐1/2 kinase demonstrated no effect upon either cytokine release or *miR‐145* expression (Fig. 2G,H,I). In contrast, inhibition of the p38 MAP kinase had differential actions upon cytokine and *miR‐145* production. Blocking p38 MAP kinase inhibited CXCL8 but not IL‐6 in both the nonsmoker and COPD ASM cells (Fig. 2J,K), and a significant increase in *miR‐145* expression was observed in the COPD ASM cells (Fig. 2L). Overall, pharmacological studies indicate that TGF‐β‐ induced *miR‐145* expression is regulated via an MEK‐1/2‐ and p38‐dependent pathway.

### The effect upon TGF‐β‐stimulated ASM cells of *miR‐145* overexpression on CXCL8 and IL‐6 release

**Figure 3:**
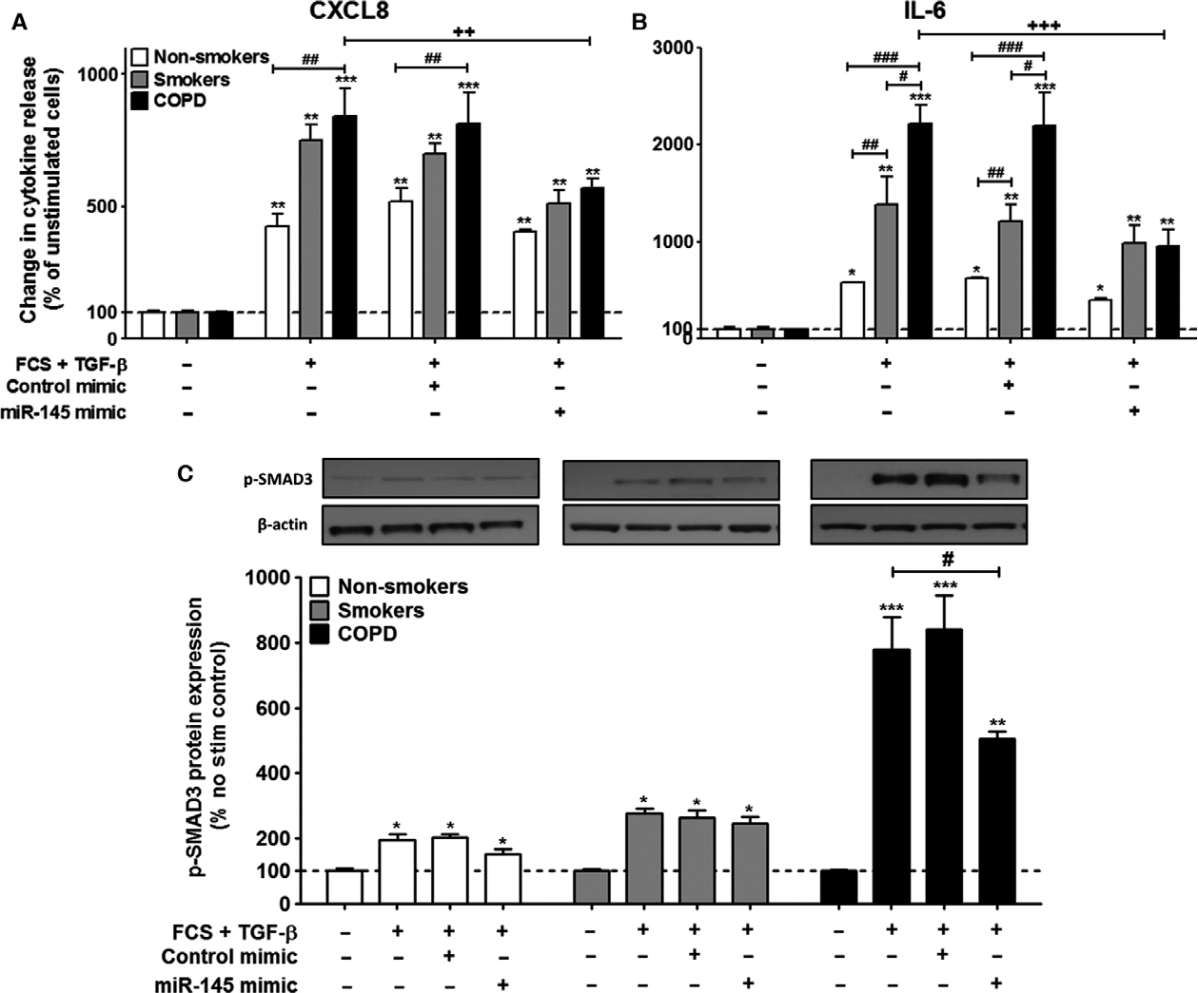
Effects of the overexpression of *miR‐145* in the ASM cells of nonsmokers, smokers and patients with COPD at 24 h. ASM cells were electroporated in the presence of buffer, control mimic or *miR‐145* mimic. Cells were then exposed to vehicle control or 1 ng·mL^−1^ TGF‐β and the release of CXCL8 (A) and IL‐6 (B) was measured by ELISA at 24 h. Furthermore, p‐SMAD3 was measure by western blotting (C). Points represent the means ± SEMs from nine ASM donors in each group. *^/#^ *P* < 0.05; **^/##/++^ *P* < 0.01; ***^/###^ *P* < 0.001.

To clarify the role of *miR‐145*, we examined the effect of overexpressing *miR‐145* on TGF‐β–induced CXCL8 and IL‐6 release. Transfection using Amaxa electroporation (Lonza, Slough, UK) showed that *miR‐145* mimics (100 nm) inhibited CXCL8 release by approximately 47% (*P* < 0.01) and IL‐6 release by approximately 49% (*P* < 0.001), to levels comparably seen in the healthy smokers (Fig. 3A,B). Altering the endogenous levels of *miR‐145* exerted no effect in either healthy or smoker ASM cells or those from patients with severe asthma. To confirm efficient transfection, we undertook parallel studies that examined the effects of a small, interfering RNA (100 nm) targeted to *IL‐6*. As demonstrated previously 12, 41, we showed a reduction in IL‐6 release induced by TGF‐β stimulation in ASM cells (data not shown), with no effect upon cell viability (data not shown).

### Effects of *miR‐145* on SMAD3

We next determined whether *miR‐145* could regulate SMAD3 phosphorylation. As previously demonstrated 33, TGF‐β (1 ng·mL^−1^) increased SMAD3 phosphorylation in the nonsmoker ASM cells at 24 h (*P* < 0.05) (Fig. 3C). For the first time, we demonstrate that there is a slight increase in phosphorylation in the non‐smokers, and an even greater degree of phosphorylation in the ASM cells from COPD patients induced by TGF‐β (*P* < 0.001) (Fig. 3C). The *miR‐145* mimic (100 nm) decreased the TGF‐β‐induced phosphorylation of SMAD3 in the COPD ASM cells by approximately 50% (*P* < 0.05) (Fig. 3C), and demonstrated no effect upon either the nonsmoker or smoker ASM cells.

## Discussion

**Figure 4:**
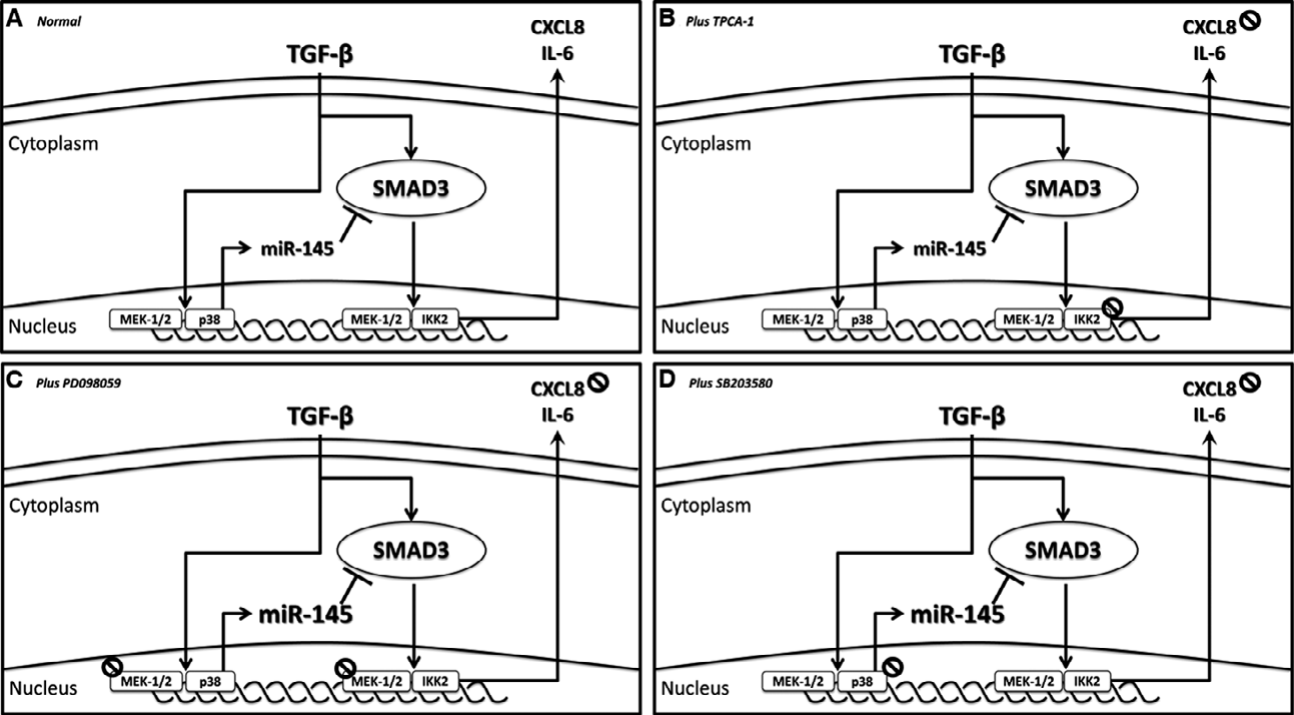
SMAD3 dependent regulation of IL‐6 & CXCL8 release by TGF‐β‐induced *miR‐145* expression. In response to TGF‐β stimulation, MEK‐1/2 and p38 activation results in increased *miR‐145* expression. The concurrent increase in expression and phosphorylation of SMAD3, is regulated by *miR‐145* to prevent further generation and release of IL‐6 & CXCL8.

We have made several important observations regarding the behaviour of ASM cells from patients with COPD. First, we showed that TGF‐β increased both ASM IL‐6 and CXCL8 release in the COPD patients to a greater degree than those from the nonsmoker subjects. We also observed a concurrent increase in the expression of both *SMAD3* and *miR‐145* in the ASM cells from the COPD patients. We next investigated the mechanisms that regulate the expression of *miR‐145*. We showed that expression of *miR‐145* is mediated, at least in part, through activation of MEK‐1/2 and p38 in ASM cells from COPD patients. Examination of the effect of these MAP kinase inhibitors upon generation of inflammatory mediators showed that IL‐6 release was mediated via IKK2 and MEK‐1/2 while CXCL8 release was mediated via IKK2, MEK‐1/2 and p38 in both the ASM cohorts, from nonsmokers and those with COPD. Finally, we found that *miR‐145* regulates the enhanced IL‐6 and CXCL8 release seen in the ASM cells from patients with COPD, that could be partly through the control of *SMAD3* (Summarized in Fig. 4).

Previous observations have described the effect of inducing hyperproliferation of ASM cells with TGF‐β in both asthma 11, 12 and COPD 44. This is the first time that TGF‐β has been shown to induce both IL‐6 and CXCL8 release from primary ASM cells isolated from individuals with COPD. The regulation of smooth muscle cell phenotype has previously been shown to be correlated with expression of *miR‐145*. Specifically, *miR‐145* has been demonstrated to regulate smooth muscle cell fate 26, 45, the contractile phenotype of VSMCs 27, 46, and acts a novel VSMC phenotypic marker in murine models 28, and prevent vein graft disease in rabbits 29. Interestingly, exposure to cigarette smoke has been shown to affect expression levels of *miR‐145* in the lungs of rats 30, and has been found to be differentially expressed in lung homogenates in rats with COPD 47. Furthermore, *miR‐145* (along with others) has recently been proposed to be a promising plasma based biomarker for the diagnosis of COPD 48. This is the first time that a role for *miR‐145* in the ASM cells from COPD patients has been reported. We have shown that expression of *miR‐145* is through activation of both MEK‐1/2 and p38. Interestingly, Hu *et al*. 49, have reported that activation of MEK‐1/2 suppresses *miR‐145* expression in VSMCs, and Kent *et al*. 50, demonstrate that *miR‐145* expression is inhibited through activation of the MAPK and JNK pathways in colorectal cancer. Furthermore, in cardiomyocytes, the protective activity of *miR‐145* is associated with modulation of both MEK‐1/2 and JNK 51 and in gastric mucosal epithelial cell regulation of *miR‐145* involves JNK 52. Similar to our results, p38 has previously been shown to be linked to *miR‐145* induction in VSMCs 53 and Hong *et al*. 54, suggest that the p38 MAPK signalling pathway promotes miRNA biogenesis by facilitating the nuclear localization of p68. Clearly, the regulation of *miR‐145* is, unsurprisingly, cell type specific.

Finally, we examined the effect of increasing *miR‐145* expression in ASM cells from COPD patients upon IL‐6 and CXCL8 release. Although studies have suggested a correlation between *miR‐145* expression and IL‐6 & CXCL8 release 55, 56, 57, 58, we show for the first time that increasing the expression of *miR‐145* can reduce the levels of IL‐6 & CXCL8 release from the COPD ASM cells to levels comparable to that of the nonsmoker ASM cells. *miR‐145* is proposed to target *SMAD3* in systemic sclerosis (SSc) 59, cystic fibrosis 23, cartilage dysfunction 60, nasopharyngeal cancer 61 and in hypertrophic scar fibroblasts (HSFBs) 62, we show here that this may also be the case in ASM cells from patients with COPD.

Interestingly, *miR‐145* is also likely to be important in regulating ASM cells in asthma, as it is highly expressed in the healthy lung 21 and in healthy ASM cells specifically 22, and inhibition of *miR‐145* inhibits eosinophilic inflammation, mucus hypersecretion, T_H_2 cytokine production and airway hyperresponsiveness in house dust mite‐induced allergic mouse airways 63.

In conclusion, *miR‐145* is vital in controlling the increased inflammatory response of human ASM cells in patients with COPD. This finding may open a new avenue in COPD therapeutics by targeting of *miRNA‐145* and diagnosis by its detection.

## Author contributions

MP and LL were responsible for preparation of the manuscript; LL, KS, IP and BT conducted *in‐vitro* experiments; KD, AH and KFC provided the primary ASM cells; MP designed the study.
